# Exploring the qualities and adaptive professional capabilities required of physicians for disaster response through perspective transformation among physicians in Fukushima, Japan

**DOI:** 10.3389/fmed.2026.1838420

**Published:** 2026-09-03

**Authors:** Megumi Yasuda, Rintaro Imafuku, Kaho Hayakawa, Maham Stanyon, Takuya Saiki

**Affiliations:** 1 Division of Medical Education, Graduate School of Medicine, Gifu University, Gifu, Japan; 2 Center for Medical Education and Career Development, Fukushima Medical University, Fukushima, Japan; 3 Nursing Research Promotion Center, Graduate School of Nursing, Nagoya City University, Nagoya, Japan; 4 Medical Education Development Center, Gifu University, Gifu, Japan

**Keywords:** adaptive professional capabilities, disaster medicine, medical education, professional identity, transformative learning

## Abstract

**Background:**

The Great East Japan Earthquake and the Fukushima Daiichi Nuclear Power Plant accident confronted health care professionals with profound uncertainty and ethical dilemmas. However, it remains unclear how physicians made sense of these extreme experiences, reconsidered their assumptions, values and professional roles, and came to recognize and embody the qualities and adaptive professional capabilities required for disaster response.

**Aim:**

Through the narratives of physicians in Fukushima, Japan, this study explores the qualities and adaptive professional capabilities required for disaster response, the processes through which they are formed, and how changes interpreted through the lens of perspective transformation are reflected in later professional practice.

**Methods:**

Semi-structured interviews were conducted with 15 physicians who experienced the Great East Japan Earthquake in Fukushima Prefecture, Japan. Interview transcripts were analyzed using inductive thematic analysis and interpretation informed by transformative learning theory. Hoggan’s three dimensions of transformation (depth, breadth, and relative stability) guided evaluation of the reported changes.

**Results:**

Physicians described confusion and conflict when confronted with situations lacking definitive answers or when professional responsibilities clashed with personal safety. These experiences prompted dialogue and critical reflection on their values and professional roles, consistent with disorienting dilemmas. Through this process, they recognized essential qualities and adaptive professional capabilities for disaster response, including crisis-oriented communication and collaboration, applying a comprehensive community-oriented perspective, recognition of one’s role and limits and selection of optimal action, embracing diverse values and contextual backgrounds, and responsiveness to uncertainty with flexible adaptability. Participants described shifts from viewing risk communication as one-way information transmission to understanding it as dialogue, and from focusing solely on individual patients to engaging with the community as a whole. Participants described these changes as continuing to inform their subsequent clinical and educational practice.

**Conclusion:**

Participants described changes in their values and professional identity that were interpreted as consistent with perspective transformation. The qualities and adaptive professional capabilities formed through reflection and dialogue under extreme circumstances became internalized in practice. These findings suggest the need to design medical education that treats uncertainty and ethical dilemmas as core learning opportunities supporting adaptive professional capabilities and ongoing professional growth.

## 1 Introduction

Across the globe, climate change and the spread of emerging infectious diseases are driving ever more frequent compound and large-scale disasters, making the maintenance and strengthening of health care delivery systems an urgent priority (1, 2). In addition to technical medical skills, non-technical skills—including the ability to make decisions under resource constraints, communicate and collaborate across professions, and adapt flexibly to rapidly changing situations—have been identified as critical determinants of the success or failure of the medical response in disaster settings (3, 4). However, although accepted in disaster medicine literature, the term “non-technical skills” may inadvertently diminish the perceived importance of what they positively contribute to professional practice. Moreover, responding to complex disaster situations requires not only discrete skills but also broader professional capabilities that enable physicians to exercise judgment, negotiate uncertainty, and respond flexibly to evolving social and medical needs (5). For this reason, we propose the term “adaptive professional capabilities” as a more constructive framing, emphasizing the integrative, context-sensitive and professionally situated nature of these competencies.

The Great East Japan Earthquake and the Fukushima Daiichi Nuclear Power Plant accident in 2011 confronted health care professionals with complex challenges that could not be addressed solely by conventional medical expertise and approaches (6). In the acute phase, widespread infrastructure disruption and shortages of medical resources required rapid decision-making and coordinated responses to large numbers of casualties (7, 8). In addition, the chaos of evacuations and population displacement following the nuclear accident caused chronic health effects and psychological distress, making ongoing involvement in psychosocial care and long-term support essential for health care professionals (8–10). Furthermore, faced with the invisible risk of radiation exposure, physicians were required to address residents’ anxieties amid scientific uncertainty while building trust through effective risk communication (11, 12). Taken together, these experiences underscored the critical importance of adaptive professional capabilities, requiring health care professionals to make composed judgments under conditions of limited resources and information, collaborate across professions, and adapt flexibly to evolving circumstances (3, 13, 14).

Existing research on disaster response has emphasized the importance of non-technical skills—such as adaptability under harsh conditions, leadership, communication, and decision-making—among specialist teams, including Disaster Medical Assistance Teams (DMATs) (15–17). However, much of this research has focused on disaster medicine specialists (3), and insufficient attention has been paid to physicians working in medical institutions in disaster-affected areas—specifically, the psychological conflicts they experienced under the extreme conditions of an unexpected compound disaster, how they navigated those conflicts, and how they reconstructed their understanding and professional values through that process.

This perspective highlights the importance of understanding how physicians develop adaptive professional capabilities through experience, particularly when confronted with situations that challenge their existing assumptions and professional roles. To understand changes in physicians’ perceptions and behaviors under such extreme circumstances, it is therefore necessary to look beyond the acquisition of knowledge and skills and instead examine the learning processes through which experiences of disaster—characterized by unpredictability and profound uncertainty—challenge physicians’ underlying beliefs and values and prompt their reconstruction.

Transformative learning theory (TLT), proposed by Mezirow, provides a framework for understanding how individuals critically examine dilemmas arising from unexpected or crisis situations, reassess their underlying assumptions, and construct new meaning (18, 19). Transformative learning may be initiated by what Mezirow termed a ‘disorienting dilemma,’ an experience that cannot be adequately addressed within existing frames of reference. Through critical reflection and dialogue with others, learners reconsider their assumptions and values and may reconstruct their frames of reference toward more inclusive and integrative perspectives.

Mezirow (20) further conceptualized perspective transformation as a 10-phase process beginning with a disorienting dilemma and progressing through self-examination, critical assessment of assumptions, exploration of new roles, planning of action, acquisition of knowledge and skills, provisional testing of new roles, development of competence and self-confidence, and eventual reintegration of a revised perspective. Building on Mezirow (20) and related TLT literature, Hoggan (21, 22) proposed depth, breadth, and relative stability as criteria for evaluating the extent of transformative learning.

Experiences of disaster are not merely transient events or temporary shifts in perception; rather, they may exert long-term influence on health care professionals’ practice and engagement with their work beyond the acute phase (23). Therefore, learning triggered by disaster may not be limited to temporary insights among disaster response specialists, but may also be sustained in the clinical and educational practices of physicians who were already working in affected regions prior to the disaster. Clarifying how physicians who experienced such disasters transformed their perceptions and values through extreme circumstances, and how those transformations have been embodied in subsequent clinical and educational practice, may provide important insights for medical education grounded in key lessons from disaster medicine.

This study aims to explore how physicians in Fukushima who experienced the 2011 compound disaster retrospectively interpreted their experiences and how these experiences prompted perspective transformation. Drawing on their narratives, the study examines how these transformations contributed to the re-evaluation of the qualities and adaptive professional capabilities required of physicians for disaster response and how these perspectives have been sustained in subsequent clinical and educational practice.

The research questions (RQ) are as follows:

RQ1: What qualities and adaptive professional capabilities do physicians in Fukushima who experienced the Great East Japan Earthquake believe are required of all physicians?

RQ2: Through what processes of perspective transformation were these qualities and capabilities formed?

RQ3: How have these perspective transformations been sustained in physicians’ clinical and educational practice?

## 2 Materials and methods

### 2.1 Research approach and design

This study was grounded in the TLT framework described above and aimed to clarify how physicians, through their experiences of disaster, underwent perspective transformation and came to recognize the qualities and adaptive professional capabilities required for effective disaster response. Because processes of transformation are deeply embedded within individual narratives, it was necessary to carefully examine physicians’ lived experiences and interpret the meaning transformations inherent in those accounts. In light of this, the study adopted a qualitative research approach grounded in an interpretive paradigm and employed a descriptive and exploratory design for data collection and analysis.

### 2.2 Data collection

Eligible participants were physicians who were already working at medical institutions in Fukushima Prefecture when the Great East Japan Earthquake occurred on March 11, 2011, and who were subsequently involved in response activities related to the earthquake and the Fukushima Daiichi Nuclear Power Plant accident. Participants were recruited directly by the research team or through recommendations from physicians familiar with disaster response activities in Fukushima. Fifteen physicians participated in the study (13 men and 2 women). At the time of the earthquake, their years of clinical experience ranged from 7 to 35 years (mean 18.5 years; median 18 years), and they were employed at major regional hospitals or university hospitals. Participants represented a range of clinical backgrounds, including emergency medicine, radiology, family medicine, obstetrics and gynecology, pediatrics, surgery, and public health.

Semi-structured interviews were conducted in person with participants between December 2023 and July 2024. All interviews were conducted in Japanese by the first author at locations chosen by the participants. Each interview lasted between 27 and 73 min, and all conversations were audio-recorded using a digital voice recorder with participants’ prior consent. The recordings were transcribed verbatim and used as textual data for analysis. During the interviews, the first author avoided suggestive or evaluative responses and used follow-up questions primarily to clarify or further explore participants’ own accounts.

The interviews explored participants’ activities on the day of the earthquake and in the days that followed, the difficulties and dilemmas they encountered and how they responded to them, and the perceived influence of their disaster response experiences on their current professional practice, including educational practice. Participants were also asked about the qualities and adaptive professional capabilities required of physicians in disaster response and the types of undergraduate and postgraduate education that might support their development, including approaches that could be incorporated into education during non-disaster periods. The interview guide was developed by the first author with reference to previous qualitative studies on competencies required in disaster medicine (15, 24), and its content was reviewed by the research team for relevance and clarity. The semi-structured interview guide was used flexibly, allowing participants to elaborate on their experiences and the interviewer to follow issues raised in their narratives. The interview questions were not structured according to TLT or Mezirow’s phases.

This study was approved by the Research Ethics Committee of the Gifu University School of Medicine and the Ethics Committee of Fukushima Medical University (Approval Nos. 2023-168 and GS2023-058).

### 2.3 Data analysis

This study initially analyzed the qualitative data inductively using a thematic analysis approach informed by Braun and Clarke’s (25) six-phase process. Initial codes and themes were generated from participants’ narratives rather than from predetermined categories derived from TLT. After the initial themes had been developed, TLT was introduced during the interpretive stage as a theoretical lens rather than as a predefined coding template. It was used to examine how participants’ disaster experiences were related to changes in their assumptions, values, frames of reference, and professional roles (18, 20), and how these changes were related to the adaptive professional capabilities identified in the analysis. The analysis involved iterative movement between the empirical patterns identified in participants’ narratives and theoretical concepts concerning disruption, reflection, changes in frames of reference, and subsequent practice. Because this interpretive process brought the empirical findings and existing theoretical perspectives into dialogue, it was abductive in nature (26, 27).

At this interpretive stage, Mezirow’s 10-phase model of transformative learning was not applied as a fixed or sequential coding framework, but was used as a conceptual lens for understanding patterns within participants’ narratives (20). In particular, this study drew on a previous study (28) that organized Mezirow’s 10 phases into broader analytic stages. Drawing on this approach, accounts of confusion and conflict arising from disaster experiences were examined in relation to disorienting dilemmas, corresponding primarily to phase 1. Narratives describing self-examination and the re-examination of assumptions, values, or standards of judgment through dialogue and reflection were interpreted in relation to critical reflection, corresponding primarily to phases 2–3. Accounts involving the reconsideration of professional roles, values, standards of judgment, or meaning frameworks were examined as potentially consistent with perspective transformation, corresponding broadly to phases 4–10. The relationships and movement across these stages were analyzed to clarify the processes of perspective transformation addressed in RQ2, while the specific themes within these transformed perspectives informed the identification of the qualities and adaptive professional capabilities addressed in RQ1. Reported changes in clinical practice, behavior, or professional stance were examined as subsequent actions related to phases 6–10 and informed the analysis of RQ3.

To distinguish pragmatic adaptation from changes potentially consistent with perspective transformation, context-specific actions, immediate behavioral adjustments made to manage disaster conditions, and the acquisition of knowledge or skills were not, on their own, interpreted as perspective transformation. Accounts were examined as potentially consistent with perspective transformation when participants described reflection and/or dialogue that led to the reconsideration of underlying assumptions, values, standards of judgment, or understandings of their professional roles. Hoggan’s (21, 22) dimensions of depth, breadth, and relative stability were used to examine the extent of the reported changes rather than to assume that all accounts represented enduring transformation of professional identity.

The verbatim transcripts were analyzed by a research team composed of five researchers with diverse professional backgrounds. The team comprised one internist (YM), one educational researcher (RI), and three medical educators (TS, KH, MS), enabling interpretation of the data from multiple perspectives. The first author, who conducted all interviews, was working at a university hospital in Fukushima and living in the region at the time of the disaster. This positionality provided contextual knowledge and may have supported rapport with participants, while also creating the possibility of shared assumptions or over-identification with particular narratives. To address this, the analysis involved researchers from different professional and geographical backgrounds, including researchers who had not directly experienced the Fukushima disaster.

All researchers first read the interview transcripts to familiarize themselves with the overall content and meaning of the data and recorded notable excerpts and analytic observations as annotations. Two researchers (YM and RI) independently conducted the initial coding. The initial codes were compared and discussed, and preliminary themes were developed by identifying broader patterns of meaning across the data. YM subsequently applied the evolving coding and thematic structure to the remaining transcripts. The full research team reviewed and refined the codes and themes, repeatedly checked their coherence against the original transcripts, and considered alternative interpretations.

Recruitment concluded when the dataset was judged to provide sufficient depth, diversity, recurrence, and relevance to address the research questions and support the central thematic structure.

Throughout the analysis, annotations were used to support interpretation and team discussion but were not treated as independent data sources. Relevant literature was used to contextualize the findings during the interpretive stage. Methodological rigor was supported through collaborative interpretation, reflexive documentation, and repeated comparison of codes and themes with the original transcripts. This study was reported in accordance with the Standards for Reporting Qualitative Research (SRQR) (29).

## 3 Results

The results are organized as follows. First, the qualities and adaptive professional capabilities corresponding to RQ1 are presented. Based on these, the respective processes of transformation related to RQ2 are described in detail. Finally, with reference to RQ3, the ways in which these transformations were manifested as concrete actions in subsequent practice are shown. An overview of the findings is presented in Table 1.

**Table 1:** The transformative learning process: from disorienting dilemma to action.

Qualities/adaptive professional capabilities	Disorienting dilemma		Critical reflection		Action
1. Crisis-oriented communication and collaboration	Communication breakdown and conflict during disaster	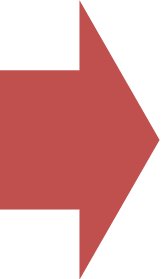	Renewed recognition of dialogue for mutual understanding and collaboration	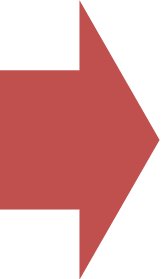	*Clinical practice* Proactive engagement with social factors amid uncertainty Systematization and practical application of disaster experience Ongoing exploration of how to be a physician in situations without clear answers *Educational practice* Interprofessional education engaging diverse community stakeholders Educational practices that deepen and expand learning from disaster experience Teaching thinking skills to persistently tackle uncertainty Educational practices that cultivate dialogue with community residents
2. Applying a comprehensive community-oriented perspective	Role confusion and community disruption		Shift from individual (micro) care to a community-wide (macro) perspective		
3. Recognition of one’s role and limits and selection of optimal action	Conflict between professional duty and fear		Reconstruction of sense of mission through dialogue and reflection		
4. Embracing diverse values and contextual backgrounds	Shifts in physicians’ values regarding the decision to evacuate		Reconsideration of professional responsibility amid diverse values		
5. Responsiveness to uncertainty and flexible adaptability	Destabilized judgment when manuals failed, and surprise at students’ initiative		Shift toward responsiveness and flexible thinking beyond manuals		

Overall, physicians experienced disruptions to their prior value frameworks—disorienting dilemmas—arising from the compound disaster of the earthquake and nuclear accident. Through reflection and dialogue (critical reflection), they reinterpreted these experiences and underwent perspective transformation, reconstructing their professional values and foundations for action as physicians. These perspective transformations were reflected in the recognition of five qualities and adaptive professional capabilities required for disaster response. More than a decade after the disaster, participants described these transformations as continuing to inform concrete actions in both clinical and educational practice.

The following five qualities and adaptive professional capabilities were identified as essential for physicians in the context of disaster response:

1. Crisis-oriented communication and collaboration
2. Applying a comprehensive community-oriented perspective
3. Recognition of one’s role and limits and selection of optimal action
4. Embracing diverse values and contextual backgrounds
5. Responsiveness to uncertainty and flexible adaptability

### 3.1 Crisis-oriented communication and collaboration

Physicians conceptualized the interpersonal and collaborative abilities required in crisis situations such as disasters as an integrated quality that enables trust-building among diverse stakeholders—including medical teams, governmental agencies, and community residents—and facilitates smooth information sharing, coordination, and consensus-building as circumstances evolve. They also recognized that, rather than unilaterally presenting professional expertise, it was necessary to develop a deep understanding of disaster-affected individuals’ anxieties and their social and psychological contexts, and to adopt a communicative stance characterized by the use of clear, accessible language tailored to the needs of the audience. During periods of confusion, two-way communication that takes into account the other person’s circumstances and psychological state was regarded as indispensable for building trust and ensuring the smooth delivery of medical care.

*“I really felt that medical communication skills as a team are so important.” (No. 4)*

*“The ability to draw people in — negotiation skills, or I guess communication skills — that’s a big one.” (No. 11)*

*“The technical stuff comes second. What’s most important, I think, is understanding the other person’s needs and situation, and figuring out how to provide the information they actually need.” (No. 12)*

#### 3.1.1 Disorienting dilemma: communication breakdown and conflict during disaster

After the disaster struck, physicians faced situations in which information sharing—both within and outside medical institutions—became difficult, and breakdowns in communication interfered with the delivery of care. Amid this confusion, physicians recognized that insufficient communication within teams had led to failures in coordination.

*“In the end, it’s about communication. If that doesn’t work, [team medicine] doesn’t work. At that time, there was no communication at all.” (No. 6)*

In addition, difficulties arose in communicating with residents. Particularly when providing information about radiation risks, even when physicians offered explanations grounded in scientific evidence, some residents prioritized information sources they personally trusted, and mutual understanding often failed to materialize.

*“Ultimately, they were getting one-sided information [from social media, etc.] and, to put it bluntly, it was like a religion. They were already kind of brainwashed, and there was no changing that.” (No. 9)*

Furthermore, faced with a chaotic situation in which conflicting information was circulating and people’s opinions diverged, another physician described feeling acutely aware of their own lack of communication skills and spoke of struggling deeply with how to convey accurate information in an understandable way.

*“I struggled because I didn’t really have the skills. I kept thinking, how can I explain this in a way that’s easy to understand? There was just this overwhelming sense of chaos—like, why is everyone thinking and saying completely different things?” (No. 12)*

#### 3.1.2 Critical reflection: renewed recognition of dialogue for mutual understanding and collaboration

Physicians recognized that, in disaster response, it was essential for professionals from different disciplines to share their knowledge and experience and to engage in dialogue in order to enhance the quality of collaboration. In activities at evacuation shelters, each profession explained the situation from its own standpoint and exchanged necessary information while responding flexibly. Through this kind of reciprocal communication, physicians came to recognize forms of collaboration that transcended professional boundaries.

*“Exchanging information and communicating with nurses, pharmacists, and the administrative staff was really important. The administrative people were working incredibly hard too, and I learned a lot from that.” (No. 4)*

At the same time, physicians came to understand that in highly uncertain disaster contexts, one-way information delivery was an insufficient response, and that two-way communication grounded in mutual understanding was indispensable.

*“Instead of just telling them what we think, we should be listening to what [the residents] are saying. I realized there’s nothing good that comes from us just being at odds with each other. … The content didn’t really change, but once I changed [my own] attitude, they [gradually] started to accept [me] a bit more.” (No. 9)*

### 3.2 Applying a comprehensive community-oriented perspective

Through their experiences of collaborating with governmental agencies in the wake of the disaster, physicians came to feel that it was necessary to grasp the needs of the community as a whole and to conceptualize medical care from a broader perspective. They also recognized the importance of viewing not only individual patients, but the community and its residents as a whole, in a comprehensive and long-term manner.

*“There really aren’t many people who have that kind of community medicine perspective. I happened to get to work with local government because of the disaster, so I was lucky to have that experience—but normally you go through your career without that. I guess it’s better to have that kind of perspective.” (No. 8)*

*“What I realized after the earthquake was that history gets cut off, family relationships get severed, whole communities get destroyed. Post-disaster care is really difficult. I found myself thinking, maybe it’s good to take a long-term view, maybe it’s good to stay involved.” (No. 2)*

#### 3.2.1 Disorienting dilemma: role confusion and community disruption

After the disaster struck, physicians were required to collaborate with governmental authorities with whom they had previously had limited interaction, and they experienced confusion regarding their own roles and positions. As they took on responsibilities that differed from their usual pre-disaster duties, they found it difficult to reconcile their professional responsibilities as physicians with administrative responses.

*“At first, [when it came to working with local government,] I was even told I wasn’t really needed. But once the nuclear accident happened, [we had no choice but to deal with] things like how to secure water, how to evacuate the children in [a particular] area, how to screen people in the community for thyroid problems.” (No. 8)*

Furthermore, physicians were confronted with the reality that rapid transformations in the community due to wide-area evacuation—including sudden population aging and social isolation—had serious impacts on residents’ health, leading them to acutely recognize the limitations of conventional individual-based medical care and the fragility of the community health care system.

*“One thing was that aging progressed all at once [because of the evacuations after the radiation accident]. And also, when a community gets fragmented like that, you start to realize just how much social frailty can negatively affect people’s health.” (No. 2)*

#### 3.2.2 Critical reflection: shift from individual (micro) care to a community-wide (macro) perspective

Physicians contrasted the sense of accomplishment gained through daily clinical practice (micro) with the broader demands of disaster response (macro), and came to reframe the necessity of a macro-level perspective focused on maintaining and rebuilding the community health care system during disasters. They also noted the lack of education related to this kind of systems thinking.

*“Disaster medicine is really about thinking how to protect the community. But for doctors, the joy of helping the patient right in front of you is bigger. Thinking about the whole system—that’s not really covered much in education, and there aren’t many chances to actually practice it either, right?” (No. 8)*

Moreover, witnessing the fragmentation of communities and the hardships faced by residents led physicians to acutely realize that individual-level care alone was insufficient. They recognized the importance of adopting a comprehensive perspective that surveys the entire community and enables simultaneous responses to multiple health challenges.

*“It made me think that it’s not just the person right in front of me—there are probably lots of other people out there struggling too.” (No. 2)*

### 3.3 Recognition of one’s role and limits and selection of optimal action

Physicians recognized the necessity of accurately understanding their own roles and limits in crisis situations in order to select the most appropriate course of action. This skill was described as encompassing objective situational judgment to achieve the best possible outcomes within the limits of one’s knowledge and skills, resilience in the face of psychologically demanding conditions, and practical decision-making that balances professional responsibility with one’s own safety.

*“I’ve always thought that doing what you can with what you actually have… that awareness itself is probably a non-technical skill.” (No. 7)*

*“What’s needed during a disaster is the ability to strategize how to achieve the best possible outcome with the resources you have, and the capacity to endure and overcome a mentally tough environment.” (No. 9)*

*“I guess it’s about figuring out where the line is—where even a doctor has no choice but to evacuate. If there are still people there, then you have to stay. How you find that middle ground—that’s probably the most important thing in a disaster.” (No. 1)*

#### 3.3.1 Disorienting dilemma: conflict between professional duty and fear

Physicians struggled with a conflict between fear of the unknown radiation risk and their responsibilities as medical professionals. They described being forced, under conditions of profound uncertainty, to decide whether to evacuate or remain at their posts.

*“It would be a lie to say I didn’t want to run away. But at the time I thought, ‘I guess we have to.’” (No. 8)*

*“[One doctor] said, ‘We could all just split up and evacuate, right?’ But I thought, if I leave now, I’ll probably never be able to practice medicine again. It would haunt me. … So in the end, I felt like there was no choice but to stay.” (No. 7)*

#### 3.3.2 Critical reflection: reconstruction of sense of mission through dialogue and reflection

In the process of working through this conflict, physicians revisited their sense of professional mission and reconsidered how they perceived and positioned it. Dialogue with radiation experts and discussions within their organizations became turning points, helping them regain composure grounded in scientific knowledge and think more constructively.

*“I don’t really remember the details [of what was explained]. But seeing all the staff focused on the same thing, I started to think, maybe there is actually a strategy for fighting this. So I thought, this isn’t the time to despair—if I don’t do something, I’ll regret it for the rest of my life.” (No. 7)*

*“[The expert] told us, ‘Calm down. This is fate. Just figure out what you need to do right now and tackle it one step at a time.’ … Everyone went quiet, but then it was like, ‘Yeah, that’s right.’ That was what got the engine going and helped us get moving.” (No. 9)*

Some physicians also came to recognize their sense of role and responsibility as physicians more fully through dialogue with family members.

*“I told [my father], ‘I kind of want to run away too.’ And he said, ‘Doctors are the last ones to run.’ He told me off, saying, ‘What are you talking about?’ … And I said, ‘Yeah, sorry.’ I figured I’d better get back to work.” (No. 1)*

Physicians also spoke about a strengthened awareness of their role in the disaster-affected area, recognizing themselves as contributors to the community’s health care.

*“At the time, there really wasn’t anyone in Fukushima who wanted to take this on. So in a way, I felt like it had to be me. I thought maybe this is why I’m here. Feeling that I was actually useful—maybe that’s what gave it meaning.” (No. 7)*

### 3.4 Embracing diverse values and contextual backgrounds

Physicians came to recognize that, in disaster response, patients, residents, and even fellow health care professionals hold differing values and positions. They recognized that, in order to respond appropriately to each person’s background and circumstances, it is essential to adopt a stance of understanding and respecting others’ choices and ways of thinking.

*“Even if people go through the exact same event, each person’s reaction to it, or what troubles them about it, can be completely different. If you don’t take that into account, you probably can’t solve anything. Everyone has their own story. Even in the same disaster, it’s absolutely different for each person.” (No. 14)*

*“I want us to develop a stance where we respect each other’s positions and ways of thinking, avoid blaming, and work together looking to the future.” (No. 5)*

#### 3.4.1 Disorienting dilemma: shifts in physicians’ values regarding the decision to evacuate

As a result of the nuclear accident, health care professionals themselves were forced to confront the serious decision of whether to evacuate. In some cases, family or social circumstances compelled individuals to act in ways that did not fully align with their own intentions, and physicians sought to understand the positions and decisions of such colleagues.

*“Some doctors had family in other prefectures who told them, ‘You can’t go to Fukushima,’ so they ended up not being able to come. And we were saying, well, I guess you can’t really blame them [for evacuating].” (No. 5)*

Tensions and friction sometimes arose between those health care professionals who evacuated and those who remained. One physician described how, even in such circumstances, they respected the site leader’s decision to permit evacuation and to explicitly affirm that the choices of those who left would be respected.

*“It’s really unfortunate that friction arises [because some evacuated and some didn’t] even though no one is at fault. I really respected that [the leader] made it clear from the start: ‘We won’t stop anyone from evacuating, and if you’re able to come back, we’d want you to work here again.’” (No. 14)*

#### 3.4.2 Critical reflection: reconsideration of professional responsibility amid diverse values

In the course of disaster response, physicians came to recognize the importance of not only attending to patients’ own wishes and concerns, but also understanding the diverse feelings and values underlying their situations—shaped by family, environment, and individual circumstances—and engaging with them in ways that respected those contexts.

*“Patients have their own thoughts and feelings, of course, but there are probably thoughts and feelings beyond just what the person says—from the people around them, their environment, their family—and each brings its own perspective.” (No. 14)*

Furthermore, through the harsh experience of the disaster, physicians also came to believe that professional responsibility should not necessarily entail excessive self-sacrifice. Consequently, they recognized the importance of understanding the circumstances behind colleagues’ decisions to leave their posts and of viewing those choices with tolerance.

*“It wasn’t that they ‘ran away.’ They had no choice but to move. Even if they wanted to stay, they couldn’t. They wanted to help—everyone did. But the situation [after the nuclear accident] just did not allow it.” (No. 5)*

### 3.5 Responsiveness to uncertainty and flexible adaptability

Through their experiences of disaster response, physicians recognized the importance of being able to think flexibly and act responsively amid rapidly changing circumstances. Faced with situations that could not be managed by manuals or existing knowledge alone, they came to believe that it was necessary to calmly accept the reality before them and to thoroughly think through the situation themselves to seek the best possible course of action.

*“You have to deal with things that weren’t planned for—things nobody even imagined would happen.” (No. 3)*

*“Adaptation itself is a kind of evolution. I think one really important quality is being able to keep striving to adapt to all the changes happening right in front of you—even if it’s really tough, even if it’s messy.” (No. 7)*

*“Rather than just technical skills, it’s about asking ourselves: What can we actually do to respond to this disaster? What can we do to help?” (No. 10)*

#### 3.5.1 Disorienting dilemma: destabilized judgment when manuals failed, and surprise at students’ initiative

In clinical settings, pre-prepared disaster response manuals did not function adequately, and physicians found themselves confronting a succession of unforeseen challenges, experiencing intense disorientation and confusion in the face of ongoing uncertainty.

*“We had manuals, sure. But we didn’t really have the know-how, so it was like — what are we supposed to do?” (No. 9)*

*“No one had any measures in place for what to do if radiation leaked outside the plant. Nobody had even imagined that scenario—we’d never considered something like that.” (No. 13)*

Amid this confusion, physicians observed medical students independently initiating volunteer activities and acting flexibly outside conventional structures, and they described feeling both surprised and hopeful.

*“[After the earthquake] the students were already taking action on their own. They set up their own volunteer groups and went around asking different places if there was anything they could help with. I remember watching them and feeling incredibly reassured.” (No. 2)*

Even physicians who would ordinarily make critical remarks about “today’s young people” found themselves moved by the initiative and flexibility shown by the younger generation in this extreme situation.

*“It’s kind of a cliché to moan about ‘young people these days,’ but at that time, I realized that when faced with a situation like this, they step up and really work hard, really paying attention to what’s going on. … Medical professionals really show incredible strength in moments like that. And honestly, the administrative staff do too—they really show amazing capability in disasters.” (No. 15)*

#### 3.5.2 Critical reflection: shift toward responsiveness and flexible thinking beyond manuals

Physicians came to recognize more fully the importance of being able to gather accurate information, analyze it calmly, make logical judgments, and reach timely decisions within conditions of limited resources.

*“In reality, there wasn’t much we’d actually managed to prepare in advance—the manuals included. So what we ended up doing was using whatever we had on hand and responding on the spot. That ability was actually pretty useful this time. … When there’s no manual, I think what really matters is being able to build a logical case, justify what you’re doing, and have the courage to act.” (No. 9)*

*“You need the ability to gather information and analyze it even in uncertain situations, think through what the best course of action is at that moment, and then rethink it again when new information comes in.” (No. 5)*

Furthermore, through witnessing the proactive actions of students and younger health care professionals, physicians came to recognize the importance of accepting changing circumstances and continually thinking for oneself about what the best course of action might be.

*“When the students said they wanted to start volunteering on their own, they were really thinking about what they themselves could do. … You never know what kind of disaster will happen, or when, or how. But in the end, you just have to think it through yourself.” (No. 10)*

*“It’s tough, but you have to respond to what’s happened. And then it’s about how you can steer it toward a better outcome. You can’t undo what’s already happened. … If you are going to respond, then you keep thinking hard about how you can move things in a better direction from here.” (No. 12)*

### 3.6 Actions in clinical practice

#### 3.6.1 Proactive engagement with social factors amid uncertainty

Physicians perceived that their disaster response experiences had cultivated an ability to act swiftly and proactively when confronted with unknown threats. Some physicians explained that their earthquake experience served as a foundation for future decision-making, particularly during the early stages of the COVID-19 pandemic, when many health care professionals hesitated in their response. This enabled them to engage diverse stakeholders, consider broader social factors, and take the initiative in responding.

*“[After the earthquake,] I had to figure out what kind of information we should start gathering, so I even went to Kobe [where they had experienced the Great Hanshin Earthquake] to learn from them. … I said, maybe Fukushima can do this now—let us conduct a prefecture-wide survey. … That’s why I did the same with COVID. I said, I’ll take that on too. What was in my mind? Well, I kept thinking, this feels similar [to the earthquake]. With it looking like so many people are going to struggle, how come no one’s stepping up?” (No. 1)*

*“I think it definitely carried over into COVID. Many of the [local government] members from the earthquake response were still involved, so those connections had expanded. If you only thought about COVID as an infectious disease issue, there’s no way you could [handle it].” (No. 8)*

Physicians also described how, drawing on their experience of harmful rumors and reputational damage after the nuclear accident, they addressed discrimination and prejudice associated with COVID-19 at an organizational level.

*“As a hospital, I feel like we were able to respond to COVID surprisingly quickly. There were also hurtful rumors during COVID. Like kids being bullied because their mum worked in the COVID ward, and so on. At that time, we set up a fever clinic as a hospital and did a lot of different things to get through it.” (No. 13)*

#### 3.6.2 Systematization and practical application of disaster experience

Physicians recognized the need to build a systematic and academic foundation rather than relying solely on their experiential knowledge. They also broadly applied the insights they had gained in real-world settings, both within and outside their institutions, including medical support for recent large-scale earthquake disasters and in-hospital disaster response training.

*“From my experience [of the earthquake], I realized there really is a properly structured field—emergency disaster medicine—and unless you study it properly and prepare yourself accordingly, there’s a big difference between the backup you can provide when you know what you are doing versus not having that knowledge… [So I started studying disaster medicine]. I actually went to Noto [in Ishikawa Prefecture] not long ago [after the major earthquake in 2024], and of course I was able to put that knowledge to use.” (No. 11)*

*“We’re now running disaster drills that really make people think, and I get the impression that staff awareness is gradually starting to change. [We need to convey] the importance of preparedness, of cultivating real people who can actually act when the time comes.” (No. 11)*

#### 3.6.3 Ongoing exploration of how to be a physician in situations without clear answers

In the wake of the disaster, as residents and patients faced diverse hardships, physicians once again recognized the importance of not being bound by the assumption that there is a single correct answer. Some continued to hold the view that a core professional responsibility of physicians is to respect individual positions and values while making objective judgments and acting flexibly.

*“It’s not about what’s right or wrong. [What matters is] understanding each person’s position and situation, and then showing objectively what should be done. That’s the part I try not to waver on.” (No. 14)*

At the same time, some health care professionals who had witnessed colleagues evacuate after the nuclear accident came to view the professional ethic of prioritizing self-sacrifice as a physician as something that is not an absolute norm. While acknowledging the importance of duty toward patients and altruism, they came to understand that imposing such expectations uniformly is not necessarily just, and that it is necessary to respect each medical professional’s own life philosophy and values.

*“It’s okay to have your own life philosophy. I mean, the idea that self-sacrifice, for the patient’s sake or whatever, has to be the number one priority—well, if that’s how someone chooses to live, that’s fine. But it’s not like you are not fit to be a medical professional if that’s not your top priority. That’s something I hope current and future health care professionals can really take on board. That said, putting profit above doing things ‘for the patient’—that’s not right either.” (No. 5)*

On the basis of such reflections, one physician described coming to accept irreversible realities of the past and continuing to seek the best possible course of action while responding flexibly, even in situations where resources are limited.

*“Take contaminated soil, for example—you can’t turn back time, right? So you just have to play the hand you’ve been dealt. … I came to think that you have to be flexible in responding to those kinds of changes.” (No. 7)*

### 3.7 Actions in educational practice

#### 3.7.1 Interprofessional education engaging diverse community stakeholders

Through their experience of the disaster, physicians came to recognize the importance of developing a comprehensive understanding of the community as a whole. In particular, having experienced first-hand the essential role of collaboration in supporting the population—not only among health care professionals but also across government, medical services, welfare services, and community residents—they became strongly aware of the need to create educational opportunities for students to learn interprofessional collaboration.

*“It’s important [for students] to understand that the medical care they see in the hospital isn’t everything—there are lots of other backgrounds beyond that. We need to deepen their understanding of community-based integrated care and help them see how medical professionals and welfare services are involved—that it’s not about who’s superior or inferior, but that everyone is working on a level playing field, each doing what they can in their own role. Once they understand that, they’ll be able to take action on their own when someone is in trouble, and not just in disaster situations.” (No. 2)*

#### 3.7.2 Educational practices that deepen and expand learning from disaster experience

Physicians deepened their recognition that, in order to prepare for the unexpected, it is crucial to continue learning knowledge and ways of thinking rather than keeping their experiences merely as personal memories. These experiences were reinterpreted as forms of learning grounded in lived reality, and physicians described the significance of extending such learning beyond the university setting to students in other contexts as well.

*“[To respond to unexpected disasters,] it’s important to keep learning solid knowledge and ways of thinking—and I feel I can convey my experiences of that time with realism and confidence [to students inside and outside the university]. When I talk about the actual example of Fukushima, it really gets across why we study and why it’s important to learn about disasters.” (No. 3)*

#### 3.7.3 Teaching thinking skills to persistently tackle uncertainty

One physician described how, with the aim of strengthening students’ ability to think things through independently in uncertain situations, they had come to position practical exercises—requiring students to apply their own knowledge and skills to explore possible responses—as an ongoing component of their regular educational practice.

*“In our practical sessions, one of the tasks is seeing how students can improvise when they’re put in situations they don’t fully understand. Another thing we focus on is encouraging them to try responding using whatever knowledge and skills they already have, adapting them as best they can. … In the end, it’s about keeping up that effort to think things through, not giving up.” (No. 7)*

#### 3.7.4 Educational practices that cultivate dialogue with community residents

Drawing on their experiences of struggling with information dissemination and communication with residents during the disaster, physicians came to strongly recognize the educational importance of expanding beyond conventional in-hospital patient communication skills and cultivating the ability to communicate effectively with residents. In particular, they placed emphasis on teaching the skills needed to explain specialized knowledge in clear, accessible language, as well as broader communication abilities that support collaboration with other professions and community members.

*“It’s really just about whether we can explain things well [to residents in the community]. So I’ve been studying training programs designed to help professionals communicate more effectively. And now we’re teaching that [to medical students].” (No. 12)*

## 4 Discussion

### 4.1 Reflection on disaster experience and transformative learning

This study qualitatively examined how physicians in Fukushima who experienced the unprecedented compound disaster of the Great East Japan Earthquake and the Fukushima Daiichi Nuclear Power Plant accident came to recognize the qualities and adaptive professional capabilities required for disaster response, and the learning processes through which these were formed. The contribution of this study lies not only in delineating the content of the qualities and adaptive professional capabilities that physicians came to recognize, but also in theoretically explicating the internal processes through which these reconstructions occurred by adopting TLT as an analytical framework (18).

In addition to the physical devastation caused by the earthquake and tsunami, the invisible and scientifically uncertain threat of radiation exposure created a sense of powerlessness among physicians, as they were unable to present a definitive “correct answer” as experts. This generated a dilemma in which they felt torn between their professional responsibilities as physicians and the safety of themselves and their families. These experiences can be interpreted as functioning not merely as confusion, but as a disorienting dilemma that fundamentally shook their professional identity as physicians. Importantly, this disorienting dilemma did not automatically lead to perspective transformation; rather, it was through subsequent critical reflection that these experiences were reinterpreted and given new meaning. Drawing on their doubts and internal conflicts regarding their own judgments and actions, physicians began to question the values, beliefs, and standards of judgment that they had previously taken for granted. This fundamental reexamination acted on their own frames of reference, ultimately leading to a renewed recognition of the qualities and adaptive professional capabilities identified in this study.

### 4.2 Transformation of physicians’ perspectives through disaster experience

The first specific transformation identified in this study was a shift in physicians’ understanding of communication. For the physicians, the dilemma lay in their experience that the assumption that residents’ anxieties about radiation risk could be alleviated through the one-way provision of scientifically grounded information—that is, the so-called “information deficit model” (30)—did not function adequately in actual interactions with residents. This experience, which can be described as a “failure of expertise” (31), appears to have prompted a departure from the existing framework in which experts unilaterally provide information, toward a new perspective in which engaging in dialogue that attends to residents’ anxieties and values is understood as a core professional responsibility. Crucially, the physicians’ dialogue functioned not merely as information sharing, but as a practical process that challenged their own assumptions and reconstructed their frames of reference. In TLT, dialogue is regarded as a catalyst for relativizing one’s assumptions through engagement with others’ perspectives (18, 20); however, the dialogue observed in this study went beyond the level of rational discourse. Rather, through repeated engagement with residents and colleagues, physicians shifted their focus toward core principles of risk communication—recognizing that asserting scientific correctness alone can inadvertently harm disaster-affected individuals (32), and that risk perception is not a matter of right or wrong but a natural emotional response (33)—and their communication evolved toward more empathetic and relational forms of dialogue. This relational dialogue (34), in which emotions and values are shared while mutual understanding is deepened, appeared to promote physicians’ reflection and to provide a foundation supporting transformative learning. Furthermore, the findings indicate that physicians’ dialogue functioned as a process through which their understanding of their professional role was gradually reorganized—from a stance centered on providing scientifically accurate explanations to one oriented toward building trust and facing uncertainty together with residents. Although this shift is consistent with the emphasis on “empathy” in the early stages of the Crisis and Emergency Risk Communication (CERC) model (35), for the physicians it was experienced not as the application of theory, but as the reconstruction of relationships emerging through practical engagement. The present study supports the view that this very process of transforming one’s understanding of professional role provides the foundation for reconfiguring risk communication as a two-way, relational mode of practice.

The next transformation identified in this study was the process through which physicians, within their social interactions that unfolded during the disaster, developed a perspective that reframed medical care from the level of the “individual” to that of “society.” As Mezirow notes, perspectives are reconstructed within dialogical relationships (20). However, confronted with the impact of community fragmentation and social isolation on residents’ health (36, 37), physicians found themselves compelled to grapple with structural issues that could not be adequately addressed through micro-level clinical interventions alone. Moreover, in practical settings where physicians collaborated with governmental authorities, nurses, care workers, and community organizations to consider resource allocation and the prioritization of support from multiple perspectives, relational dialogue emerged (34). This dialogue transcended professional boundaries and mutually illuminated how medical care is shaped by, and in turn shapes, different dimensions of society. Within these interactions, collective reflection incorporating social context was also observed (34, 38). These interrelated interactions are significant in that they functioned as a practical foundation for reorganizing physicians’ frames of reference—shifting from a view that confines medical care to the individual level toward one that encompasses the community as a whole system. Furthermore, this expansion of perspective is consistent with Worley’s concept of the social axis (39), and the conceptual framework of the social determinants of health (SDH) (40), suggesting that physicians were prompted to recognize social structures themselves as integral components of medical care.

The third transformation this study revealed was a qualitative shift in physicians’ ethical perspectives. Triggered by ethical dilemmas that arose during the disaster, physicians reconstructed their perspective from a conventional normative ethics premised on “self-sacrifice” toward a more practice-oriented ethics that seeks to sustain collaboration while respecting diverse values. The evacuation of health care professionals following the nuclear accident functioned as a disorienting dilemma in which physicians’ professional responsibilities and personal safety stood in sharp conflict (41, 42). Although the rightness or wrongness of colleagues’ decisions about whether to evacuate or remain was not openly debated, physicians demonstrated a reflective stance in which they imaginatively retraced the circumstances, fears, and values underlying those decisions. This reflection appeared as an internalized form of reflective dialogue (38), characterized not by simple judgments of right or wrong, but by an effort to understand the contextual conditions that led to particular choices. Furthermore, this process revealed a reciprocal dynamic in which understanding others led to a reexamination of one’s own ethical assumptions. This provides a complementary perspective that renders Mezirow’s notion of “empathetic understanding” in more interactive terms (20). Such multilayered reflection also resonates with Narita’s account of professionalism during the Fukushima disaster (43), which underscored the ethical complexity of decision-making and the importance of approaching others’ choices with compassion rather than blame. Taken together, these findings suggest that physicians gained an opportunity to reconstruct professionalism not as a self-sacrificial duty, but as a dynamic ethical practice grounded in mutual understanding and empathy.

### 4.3 Transformative learning and professional identity formation

In examining the nature of such perspective transformation, Hoggan states that transformation should be evaluated in terms of the depth of change, the breadth of domains to which it extends, and its relative stability over time (21, 22). The changes identified in this study extend beyond the acquisition of specialized knowledge and skills to the reconstruction of meaning frameworks—including physicians’ self-understanding, values, and underlying standards of judgment—and thus can be positioned as transformations of considerable depth, operating at the level of professional identity. This interpretation is consistent with arguments that conceptualize transformative learning not merely as cognitive change, but as a process involving the reconfiguration of self-understanding and identity (28, 44). Furthermore, the reconstruction of “how to be a physician” identified in this study can also be understood from the perspective of professional identity formation (PIF). Recent medical education research conceptualizes PIF as a dynamic process shaped not only by individual reflection but also through social interaction and participation in practice (45, 46). It posits that *being* a physician, the process of *becoming* a physician, and *belonging* within the professional community are interrelated and continually reorganized (46). In this sense, the findings suggest that the disaster was not merely an experience, but functioned as a catalyst for reconstructing physicians’ professional identity through profound reflection, driven by intense uncertainty and ethical dilemmas.

The impact of these transformations extended beyond the limited context of disaster response, permeating multiple domains of practice including routine clinical care, infectious disease response, community medicine, and educational practice. The learning outcomes functioned as a framework that guided physicians’ practice as a whole. This aspect—where transformation expands beyond the individual’s internal perspective into a reconfiguration of practice and ways of understanding the world—resonates with positions that conceptualize transformative learning as ontological and social change (47). Furthermore, the fact that these perspectives continued to be reflected in concrete actions more than a decade after the disaster indicates that the transformation was not a transient reaction, but had been internalized as a relatively stable mode of practice. Taken together, the learning outcomes identified in this study can be theoretically positioned as genuinely transformative learning when evaluated according to Hoggan’s (21, 22) criteria of depth, breadth, and relative stability.

The perspective transformation identified in this study can be understood not simply as a consequence of the unique nature of the disaster itself, but rather as the result of physicians confronting experiences marked by profound uncertainty and ethical dilemmas and reinterpreting those experiences through reflection and dialogue, thereby shaping their values and understanding of their professional roles over the long term. This point offers important implications not only for disaster response, but also for physicians’ professional development and educational practice more broadly.

### 4.4 Implications for educational practice

The findings of this study suggest that, in future medical education, it is important not merely to recreate disaster situations, but to consider the kinds of questions that physicians—whose perspectives have been transformed through disaster experience—should pose to learners. The dilemmas experienced by the participants in this study may serve as catalysts for reflection, prompting learners to reexamine their own assumptions and values.

#### 4.4.1 Dialogue-based risk communication under uncertainty

In risk communication education, it is important not only to teach techniques and models, but also to present situations for reflection in which scientifically correct explanations do not necessarily support or reassure people. For example, by examining scenarios in which well-reasoned arguments provoke resistance or distrust among residents, learners can confront the limits of persuasion and gain an opportunity to reflect on why dialogue is necessary. This question is relevant not only to disaster response but also to everyday clinical practice, such as explaining medical issues to patients in situations characterized by high uncertainty.

#### 4.4.2 The limits of medical intervention and expansion toward a social systems perspective

To cultivate a comprehensive community-oriented perspective, learning opportunities that involve reflecting on challenges that are difficult to address through medical intervention alone—such as social isolation or reputational damage—can be effective. By confronting such situations, learners may, through the tension of realizing that diagnosis and treatment alone cannot fully support patients, come to expand their understanding of medicine from the level of the individual body to that of the broader social system. This process provides an opportunity to reconsider the significance of collaboration with multiple professions and governmental actors in community medicine and public health.

#### 4.4.3 Ethical sensitivity in situations without clear answers

In ethics education, in addition to understanding norms and principles, it is indispensable to create space for dialogue that engages with conflicts for which there is no predetermined correct answer—such as situations in which professional responsibility and personal safety come into conflict. Rather than reaching definitive judgments about right or wrong, emphasizing reflective dialogue that involves imaginatively reconstructing and articulating the contexts and conflicts underlying particular decisions may foster the ethical sensitivity and tolerance toward others that are required in modern medicine, where diverse values intersect.

### 4.5 Limitations

This study has several limitations. First, the interviews were conducted more than a decade after the 2011 disaster. Participants’ accounts may therefore have been influenced by selective memory, retrospective reinterpretation, subsequent professional experiences, and later events such as the COVID-19 pandemic. At the same time, the study focused on how participants currently made meaning of their disaster experiences rather than on reconstructing the events of 2011 as contemporaneous facts. Accordingly, the findings reflect participants’ retrospective perceptions of change and cannot independently establish when these changes occurred or their continuity over time.

Second, because participants were recruited directly or through recommendations, physicians who were more willing to reflect on their experiences or more motivated to participate may have been more likely to be included. The sample may also underrepresent physicians who left the region, changed professional roles, experienced substantial psychological distress, or preferred not to revisit the disaster. Although the dataset provided sufficient depth and diversity to address the research questions, the findings should be interpreted as reflecting the experiences represented in this sample.

Third, the first author’s experience of living and working in Fukushima at the time of the disaster provided contextual understanding but may also have influenced the interviews and interpretation. Reflexive documentation, collaborative analysis by researchers with different professional and geographical backgrounds, and repeated comparison with the original transcripts were used to address this possibility.

Finally, the findings are grounded in the distinctive context of the Fukushima compound disaster. They are therefore not intended for direct generalization to other disasters, but may offer conceptually transferable insights into how physicians reconsider professional roles, values, and practices under conditions of uncertainty and ethical conflict.

Future research should focus on designing and evaluating educational programs that support the formation of these qualities and adaptive professional capabilities during non-disaster periods and that facilitate perspective transformation. Moreover, comparative examination of perspective transformation across professions beyond physicians is expected to expand the theoretical foundation of interprofessional education.

## 5 Conclusion

Through the narratives of physicians who experienced disaster response, this study clarified how the qualities and adaptive professional capabilities required in disaster response are formed through specific learning processes. Analysis based on the framework of TLT suggested that these changes extended beyond temporary adaptations and were consistent with deep and broad perspective transformations involving reconsideration of physicians’ values and understanding of their professional roles, with participants reporting that these changes continued to inform their subsequent clinical and educational practice. The findings of this study suggest the need to reconsider medical education in ways that do not isolate disaster response as a special or exceptional experience, but instead utilize experiences involving uncertainty and dilemmas as reflective resources to support physicians’ professional growth.

## Data Availability

The qualitative interview data are not publicly available because the study protocol and informed consent did not include permission for public sharing of the interview data, and the data may contain potentially identifiable information. Further inquiries can be directed to the corresponding author.
